# Inositol and Non-Alcoholic Fatty Liver Disease: A Systematic Review on Deficiencies and Supplementation

**DOI:** 10.3390/nu12113379

**Published:** 2020-11-03

**Authors:** Arianna Pani, Riccardo Giossi, Danilo Menichelli, Veronica Andrea Fittipaldo, Francesca Agnelli, Elvira Inglese, Alessandra Romandini, Rossana Roncato, Basilio Pintaudi, Francesco Del Sole, Francesco Scaglione

**Affiliations:** 1 Department of Oncology and Hemato-oncology, Postgraduate School of Clinical Pharmacology, University of Milan, 20129 Milan, Italy; arianna.pani@unimi.it (A.P.); riccardo.giossi@unimi.it (R.G.); alessandra.romandini@unimi.it (A.R.); rroncato@cro.it (R.R.); francesco.scaglione@unimi.it (F.S.); 2 Department of Neuroimmunology and Neuromuscular Diseases, Fondazione I.R.C.C.S., Istituto Neurologico Carlo Besta, 20133 Milan, Italy; 3 Department of Clinical, Internal, Anesthesiologic and Cardiovascular Sciences, Atherothrombosis Center, I Medical Clinic l, Sapienza University of Rome, 00161 Rome, Italy; francesco.delsole@uniroma1.it; 4 Oncology Department, Mario Negri Institute for Pharmacological Research I.R.C.C.S., 20156 Milan, Italy; veronicaandrea.fittipaldo@marionegri.it; 5 Internal Medicine Department, ASST Great Metropolitan Hospital Niguarda, 20162 Milan, Italy; francesca.agnelli@ospedaleniguarda.it; 6 Department of Laboratory Medicine, ASST Great Metropolitan Hospital Niguarda, 20162 Milan, Italy; elvira.inglese@ospedaleniguarda.it; 7 Experimental & Clinical Pharmacology Unit, Oncology Referral Center (CRO), IRCCS, 33081 Aviano, Italy; 8 SSD Diabetes Unit, ASST Great Metropolitan Hospital Niguarda, 20162 Milan, Italy; basilio.pintaudi@ospedaleniguarda.it

**Keywords:** non-alcoholic fatty liver disease, NAFLD, inositol, myoinositol, chiro-inositol, systematic review

## Abstract

Liver lipid accumulation is a hallmark of non-alcoholic fatty liver disease (NAFLD), broadly associated with insulin resistance. Inositols (INS) are ubiquitous polyols implied in many physiological functions. They are produced endogenously, are present in many foods and in dietary supplements. Alterations in INS metabolism seems to play a role in diseases involving insulin resistance such as diabetes and polycystic ovary syndrome. Given its role in other metabolic syndromes, the hypothesis of an INS role as a supplement in NAFLD is intriguing. We performed a systematic review of the literature to find preclinical and clinical evidence of INS supplementation efficacy in NAFLD patients. We retrieved 10 studies on animal models assessing Myoinosiol or Pinitol deficiency or supplementation and one human randomized controlled trial (RCT). Overall, INS deficiency was associated with increased fatty liver in animals. Conversely, INS supplementation in animal models of fatty liver reduced hepatic triglycerides and cholesterol accumulation and maintained a normal ultrastructural liver histopathology. In the one included RCT, Pinitol supplementation obtained similar results. Pinitol significantly reduced liver fat, post-prandial triglycerides, AST levels, lipid peroxidation increasing glutathione peroxidase activity. These results, despite being limited, indicate the need for further evaluation of INS in NAFLD in larger clinical trials.

## 1. Introduction

Non-alcoholic fatty liver disease (NAFLD) is the most important emerging liver disease, and its rising incidence in different countries seems to partly explain the new chronic liver disease pandemic. In Italy, the incidence of NAFLD is 18.5 per 1000 person/years, and due to the high number of risk factors in these patients, its prevalence can increase up to 70% in subgroups of patients affected by type 2 diabetes mellitus [1].

Non-alcoholic fatty liver disease burden does not only weigh on the liver, but is burdened by a series of cardiovascular, neurological, and kidney complications, making the treatment of this disease crucial for the improvement of both survival and quality of life in these patients. Non-alcoholic fatty liver disease patients have an increased risk of developing not only non-alcoholic steatohepatitis (NASH), but also liver fibrosis, liver cirrhosis, and hepatocellular carcinoma (HCC), albeit NAFLD, itself, being the third most common cause of liver transplantation [2]. As a consequence of the cross-talk between metabolic liver disease and the cardiovascular system, NAFLD patients carry an increased risk of cardiovascular events [3], but more specifically they develop cumulative damage in several tissues such as valves, myocardium, and conduction systems [4]. Chronic kidney disease also shows an association with NAFLD, and a more rapid decline of the glomerular filtration rate was present in NAFLD patients [5].

Several treatments are still under study in order to prevent and slow down the progression of NAFLD, targeting several steps in the pathogenesis of the disease. Starting with diet, metabolic targets, such as peroxisome proliferator-activated receptor gamma (PPARγ) [6], and going through cell stress, apoptosis, oxidative stress, and immune response [7], evidence for a structured treatment tailored to the patient are still lacking.

Inositols (INS) are ubiquitous polyols implied in many physiological functions. They are produced endogenously, are present in many foods and are available as dietary supplements. Alterations in INS absorption, metabolism and excretion seems to play a role in metabolic diseases involving insulin resistance, recently the therapeutic role of INS in these diseases is gaining more attention and showing potential benefits.

The aim of this paper was to discuss the potential role of INS in NAFLD and their potential as therapeutic agents to attenuate the metabolic cascade that leads to the progression of liver disease. We will provide a general overview of the physiopathological role of INS in humans and their pharmacological properties as well as a systematic review of the available preclinical and clinical evidence regarding INS deficiency and supplementation in NAFLD.

## 2. Biological Role of Inositol and Its Derivates

**Figure 1:**
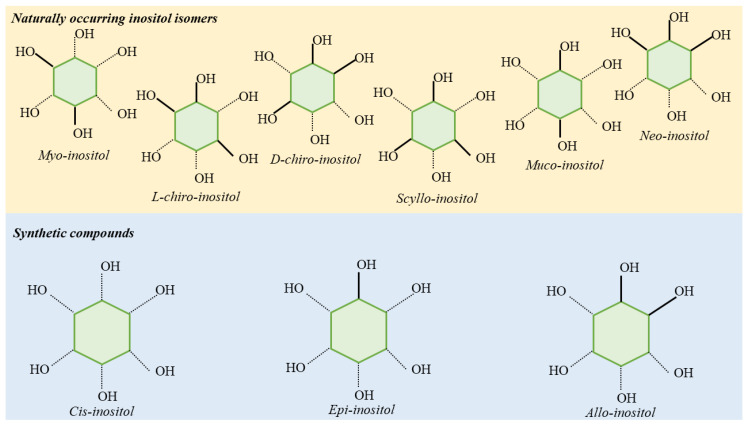
Inositol stereoisomers. Myo-, D-chiro-, L-chiro, Muco-, Scyllo-, and Neo- are the isomers naturally found in plants and animals. Allo-, cis-, and epi- stereoisomers are the synthetically obtained compounds.

Inositols are 6-carbon sugar alcohols. There are 9 INS isomers (Figure 1) differing one from the other on the basis of the spatial orientation of the hydroxyl groups.

The most common form is myoinositol (MI) which is present in animals, plant cells, and in foods [8]. Myoinositol can be found in free form, as inositol-associated phospholipid and as phosphate or pyrophosphate INS derivatives (e.g., inositol hexaphosphate or phytic acid and derived diphosphates and inositol trisphosphate). Phytic acid has a fundamental role as a storage form of phosphorus in plants. Bran and seeds are particularly rich in MI, and beans and peas contain high amounts of phytic acid as vegetables. Myoinositol can be synthetized de novo endogenously from D-glucose: this biosynthesis of MI in humans occurs predominantly in kidneys, though other tissues can also produce it. Myoinositol catabolism also takes place in kidneys, through the oxidation to D-glucuronic acid. In addition, kidneys play and important role in the regulation of INS plasmatic concentrations.

Inositol has many key functions in biological systems. It plays a very important structural role in eukaryotic cells serving as a secondary messenger, particularly in the form of inositol trisphosphate (IP3) and phosphatidylinositol phosphate lipids (PIP2 or PIP3) and INS glycans. Membrane phosphatidylinositol are fundamental in cell response to external stimuli such as hormones and neurotransmitters. The best studied mechanism of response to stimulation is the IP3-mediated cytosolic release of calcium from the endoplasmic reticulum [9]. Inositol trisphosphate is responsible for the regulation of the activity of hormones such as insulin, follicle-stimulating hormone (FSH), and thyroid stimulating hormone (TSH) [10,11,12]. Furthermore, MI is responsible for oocyte maturation and is involved also in several functions of the male reproductive system [13]. In addition to the classic insulin signaling pathway involving phosphoinositide 3 kinase (PI3K) and protein kinase B/Akt (PBK/Akt) as second messengers, a complementary pathway involving INS phosphoglycans (IPGs) exists [14]. After the stimulation of insulin, phospholipase C causes the liberation of IPGs, which acts as an insulin-mimetic secondary messenger in response to insulin stimulation, promoting GLUT-4 translocation, glucose uptake, and glycogen synthase [15].

Despite MI being the most abundant INS isoform, a second important active isomer is D-chiro-inositol (DCI), which is a derivative of MI, formed via inversion in the configuration of hydroxyls performed by the insulin-dependent enzyme NAD/NADH epimerase [16]. The epimerase acts upon insulin stimulation.

The liberation of IPGs by the phospholipase C mediates the release of IPGs containing both MI or DCI, which promotes the activation of different enzymes: DCI stimulates pyruvate dehydrogenase phosphatase, while MI inhibits protein kinase A and adenylyl cyclase [17]. Myoinositol levels are higher in tissues utilizing large amounts of glucose such as the brain, heart, and ovaries. D-chiro-inositol is more prevalent in tissues requiring glucose storage such as liver and muscles. In insulin-resistance conditions, MI epimerization is impaired in muscles, fat, and liver, and a correlation between the degree of insulin resistance and reduction in the DCI/MI ratio was observed.

Furthermore, in patients affected by insulin resistance, diabetes, and metabolic syndrome, a decreased availability of MI has been observed due to the increased MI urinary loss caused by the glucose-mediated inhibition of MI reabsorption in the kidney, while DCI urinary levels are reduced [17,18].

The role of INS in insulin resistance is still not completely understood. It is not clear if it depends on a reduction in the membrane availability of IPGs or an impairment in the epimerase activation. However, many studies have demonstrated the beneficial role of INS in conditions with underlying insulin resistance [19,20,21,22,23,24].

## 3. Inositol Pharmacology

Inositol is absorbed in the small intestine, and time plasma concentration after oral administration is obtained at 4 h (T_max_) [25]. Maximal plasma concentration (C_max_) is 36–45 mcg [26]. Inositol is actively transported by intestinal cells via a Na + dependent transporter, and glucose is able to affect this process through a non-competitive mechanism. Intestinal absorption of INS is influenced by lipids; thus, the pharmaceutical form in soft gel capsules has a better bioavailability than powder [27].

As dietary supplements, MI and DCI are well tolerated. Despite the shortage of direct toxicity studies, safety of INS supplementation in humans has been analyzed in several preclinical and clinical studies at doses ranging from 4 to 12 g/day. Gastrointestinal adverse effects have been reported in the literature with an incidence of 5% at doses of 12 g/day or higher [28]. In a limited population of patients with psychiatric disorders, mild neurological drug-related adverse effects have been observed [29]. A review of five RCTs evaluating the efficacy of INS supplementation during pregnancy for the prevention of gestational diabetes (GDM) reported a beneficial effect on GDM incidence and preterm delivery rate, with no reported adverse events (411 patients included) [30]. Furthermore, INS administration at the dose of 4 g/day has not received any reports of adverse events during clinical studies so far [31]. Inositol supplementation has shown to be effective in treating different diseases. Several studies have reported positive effects of INS supplementation in fasting blood glucose improvement and HbA1c [20,32]. Also, GDM, MI or DCI supplementation has provided encouraging results, positively affecting maternal fetal outcomes and reducing glucose variability [33,34]. Another syndrome which seems to possibly benefit from INS supplementation is polycystic ovary syndrome (PCOS), where MI demonstrated results similar to metformin in terms of homeostatic model assessment-insulin resistance (HOMA-IR) reduction and other metabolic outcomes such as BMI and menstrual cycle improvement [10,24,35].

## 4. Systematic Review Methods

### 4.1. Eligibility Criteria

We included all original clinical research articles in English with the full-text available. In particular, we included all studies that investigated the role of INS, as Mesoinositol, Myoinositol or Chiro-inositol, deficiency or supplementation in preclinical in vitro or in vivo models as well as in clinical settings in patients with non-alcoholic fatty liver disease. We did not include the following: (1) case reports, editorials/comments, letters; (2) subgroup analyses from the same clinical trial; (3) studies not addressing study questions, (4) reviews or meta-analysis.

### 4.2. Information Sources and Search Strategy

We performed a systematic review of the literature searching MEDLINE via PubMed, EMBASE, and Cochrane Library for a combination of the following keywords: “inositol”, “mesoinositol”, “myoinositol”, “chiro-inositol”, “non-alcoholic fatty liver disease”, “NAFLD”, “Nonalcoholic Fatty Liver Disease”, “fatty liver nonalcoholic”, “Nonalcoholic Fatty Livers”, “Nonalcoholic Steatohepatitis”, and “steatohepatitis nonalcoholic”. References of included articles were searched for other publications relevant to the present review. The research strategy (Supplementary Materials File S1) was performed according to PRISMA guidelines with no time restriction until 9 October 2020.

### 4.3. Study Selection

The study selection was performed in multiple phases. In the first phase, potentially relevant studies were obtained by combined searches of electronic databases using the selected abovementioned keywords. Then, studies not in English, with no abstract/full text were excluded. In the second phase, studies were reviewed and excluded by study typology; thus, letters, editorials, case reports, and comments were excluded. The third phase consisted of a detailed analysis of full-text articles to assess whether they addressed the specific study question.

### 4.4. Data Collection Process and Data Items

All authors independently screened the titles and abstracts of manuscripts identified through the database searches to identify studies potentially eligible for further assessment. For each study, we collected the following information: authors, year of publication, study typology, study aim, main results, and INS evaluated.

### 4.5. Ethical Review

Given the study type (i.e., review article), ethical approval was not necessary.

## 5. Results

**Figure 2:**
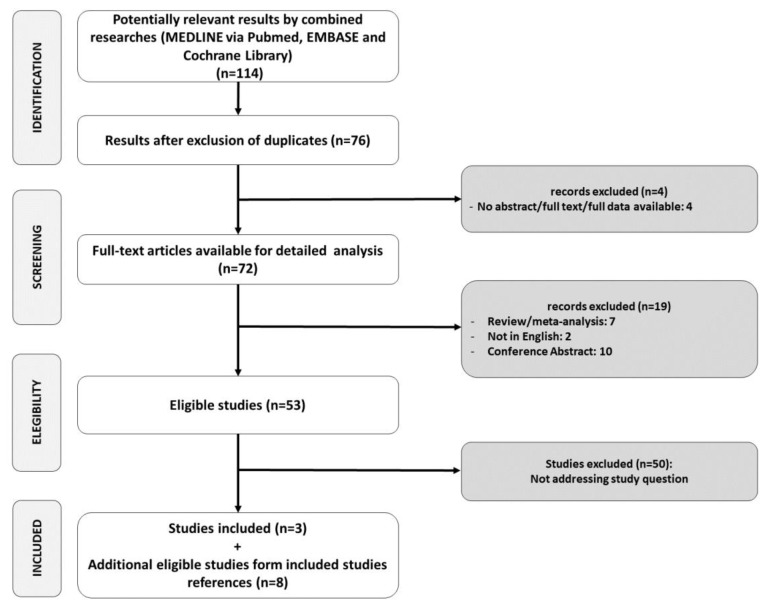
Study selection.

After duplicates removal, a total of 76 references were screened by title and abstract. Overall, 73 references were excluded with reason (Figure 2). Three studies (one animal deficiency, one animal supplementation, and human supplementation trial) were included. After the revision of references of included studies, an additional six studies assessing animal supplementation and two assessing animal deficiency were retrieved. A total of 11 studies were included in our systematic review. Included study characteristics are presented in Table 1.

## 6. Inositol Deficiency in NAFLD

Non-alcoholic fatty liver disease is now considered as a hepatic component of the metabolic syndrome and, although its incidence is still rising, no specific therapies are actually available. Over the last decades, some nutraceuticals, such as vitamin C and E, silymarin, flavonoids or resveratrol, were raised as potential treatments for NAFLD by reducing oxidative stress, inflammation, and insulin resistance [47,48,49,50]. However, these treatments counteract the pathological effects of the fatty liver but do not target specific metabolic and signal transduction pathways.

Rather, a potential preventive role on NAFLD may be represented by INS. Indeed, the lack of INS and its compounds may worsen the fatty liver disease as shown in a zebrafish model, in which a reduction of phosphatidylinositol synthesis increased endoplasmic reticulum stress and, consequently, hepatic steatosis [38]. The role of INS in preventing the development of NAFLD is enhanced in rats, where the withdrawal of INS was associated with the onset of NAFLD, especially if accompanied with a diet rich in fat [51].

Rats with MI deficiency had increased levels of triacylglycerols in the liver (2.6− and 5.3−fold higher for one and two weeks of treatment, respectively), a concomitant increase in the non-esterified fatty acid levels in serum, and raised levels of cholesterol in the liver, causing fat accumulation, compared to controls [36,37]. This condition may be reversible administrating MI and omega-3 fatty acids. In addition, MIs may reduce serum lipid levels lowering the risk of fatty liver [52].

Another fundamental role of INS in the prevention of fatty liver disease is to reduce the risk factors that cause steatosis. Indeed, MI and DCI are involved in the treatment of insulin resistance states. Indeed, both isomers have been shown to exert insulin-mimetic action and to lower postprandial glucose [53]. Of note, DCI may improve the insulin sensitivity of α-cells, which could control glucagon levels in patients with diabetes mellitus.

Furthermore, patients with uncontrolled diabetes mellitus had also polyuria increasing the excretion of INS by kidneys and worsening the insulin resistance of patients.

This evidence regarding INS deficiency and NAFLD, diabetes, and dyslipidemia lead us to study the effect of supplementation of these molecules on chronic diseases.

## 7. Inositol Supplementation in NAFLD

Compared to relatively vast preclinical and clinical research on other diseases, such as GDM, PCOS, and diabetes, only limited and introductory evidence is available on INS supplementation in NAFLD. Nevertheless, potentially relevant effects of INS in reducing liver fatty acids accumulation were enlightened by some preclinical studies in diet-induced nonalcoholic fatty liver animal models. Lipids accumulation and NAFLD, as shown in genetic and animal models [54], could activate unfolded protein response (UPR). This is a mechanism to avoid the accumulation of unfolding of proteins in the endoplasmic reticulum (ER) through the triggering of three distinct signal transduction pathways mediated by inositol requiring (IRE) 1α, PKR-like ER kinase (PERK), and activating transcription factor (ATF) 6α. These mechanisms are activated during ER stress and they promote insulin resistance and obesity in animal models. UPR also acts on numerous inflammatory pathways, including NFkB, increasing liver damage and apoptosis [55,56].

Studies on supplementation in animal models so far encompass MI, phytic acid, a six-polyphosphate form of INS, and Pinitol, a monomethylated form of DCI.

The supplementation of all these INS forms reduced liver TG, CE, and free fatty acid content [39,40,41,42,45]. Hepatic content of phospholipids was lowered by Pinitol, too [41]. Similarly, hamsters fed with a high-calorie, high-CE diet showed a reduced accumulation of white adipose tissue when supplemented with Pinitol. Pinitol reduced hepatic TG and CE, reduced plasmatic total CE and LDL, and increased HDL [43].

Hepatic activity of fatty acid synthesis enzymes was reduced by MI, phytic acid, and Pinitol [39,40]. Myoinositol reduced the expression of genes encoding enzymes for fatty acid synthesis [45]. Also, MI reduced the fructose-induced, carbohydrate-responsive element-binding protein, an important lipogenesis transcription factor, binding to genes involved in fatty acid synthesis, though not reaching statistical significance [45].

Pinitol supplementation lessened the decline in hepatic levels of vitamin E, vitamin C, and reduced glutathione and reverted the reduction in the activity of enzymatic antioxidants like catalase, superoxide dismutase, glutathione peroxidase, and glutathione-S-transferase [44].

These experiments were conducted in different animal models including rats fed with high-glucose, high-fructose or high fructose with supplementation with MI diets [45]; rats fed with high-sucrose compared to corn starch diets [40,44]; streptozotocin-induced diabetic rats [41,44] where Pinitol also exerted an anti-hyperglycemic effect. Pinitol also reduced serum TNF-α, ALT, and AST in D-galactosamine-induced hepatic toxicity in high-fat fed rats [42].

Liver histopathology showed a diminished lipid accumulation in pinitol-treated hamsters, whereas the untreated group developed a fatty liver with significantly larger adipocytes [43]. Likewise, pinitol normalized liver ultrastructure changes caused by streptozotocin-induced diabetes in rats, which caused periportal fibrosis, hepatocyte and blood vessels distortion, microvesicular vacuolization, lipid accumulation, and mitochondria and glycogen reduction compared to untreated controls [44].

These observations, albeit mainly in diabetes animal models, suggest the possibility to explore the role of INS in clinical studies involving NAFLD patients, especially considering that NASH is an important cause of disease progression in the NAFLD spectrum [57].

Recently, a double-blind, placebo-controlled, randomized clinical trial (RCT) was performed to evaluate the effects of two different doses of pinitol in 90 patients with NAFLD who were not taking medications and dietary supplements. After 12 weeks of treatment, no significant between-group differences in liver fat reduction were observed. However, there was a significant reduction in liver fat in the low dose pinitol, possibly due to the significantly lower baseline liver fat content in the high-dose group. Pinitol treatment reduced AST levels compared to placebo. The ALT and GGT levels were non-different between groups. Non-significant differences were observed in the lipid profile between groups, unless a small nonsignificant reduction in total CE and LDL was observed. These findings were accompanied by the increased of urinary malondialdehyde (a marker of lipid oxidation) in the placebo group compared to pinitol arms. The levels of glutathione peroxidase (an enzyme involved in oxidative damage reduction) increased in both pinitol arms and decreased in placebo. The authors reported no significant adverse events. In the same RCT, after a high-fat formula administration, pinitol reduced postprandial TG blood levels compared to the placebo [46]. These observations, despite being limited by a small sample size and a relatively short observation period, are encouraging to further evaluate INS supplementation efficacy and safety in NAFLD in larger RCTs.

## 8. Discussion

We here report the first literature review on the role of INS in NAFLD, evaluating preclinical and clinical evidences. Despite the shortage of quality of evidence and the need for additional clinical trials, the beneficial role of INS supplementation in NAFLD may be presumed.

One of the mechanisms proposed for the underlying positive effects of INS supplements is based on the hypothesis that the increased availability of INS could intensify the insulin pathway through an enhanced signaling mediated by IPGs [14,58]. Furthermore, INS could have a protective role against oxidative stress generated by cell metabolism [59]. Also, a positive effect on cytoskeleton regulation in cystic ovaries interacting with steroidogenesis has been observed [59]. Therefore, multiple theories explaining INS action in metabolic diseases have been proposed. However, as stated by Robert H. Mitchell, independently from the specific effect of INS, it appears clear that supplementation ensures a large availability of INS to supply central cell processes that rely on abundant INS-containing cell components [60].

Despite no specific study regarding the role of INS in NAFLD-associated HCC existing, some interesting evidence on INS and HCC has been published. A tissue chromatography-mass spectrometry (GCMS) metabolomic analysis has shown a downregulation in INS levels in HCC liver tissues compared with non-HCC livers [61]. This is the result of a tumor-induced enhanced aerobic glycolysis due to the metabolic remodeling resulting from the perturbation in the PI3K–AKT–mTOR pathway. Furthermore, another study on hepatocyte-specificTrim24-null mutant mouse model (spontaneously developing HCC), has explored the role myo-inositoltrispyrophosphate as an anticancer drug. The group of animals implemented with myo-inositoltrispyrophosphate showed no differences in terms of tumor growth except a two month improvement in overall survival [62]. Numerous studies have reported the potential of inositols as anti-tumor compounds [63], and this could be confirmed also in NAFLD-associated HCC, but further studies are needed.

Despite many clinical trials evaluating the efficacy of INS in different settings, poor quality evidence is available on the safety of INS. A meta-analysis evaluating the safety and efficacy of inositol supplementation in preterm infants for the prevention of the prematurity retinopathy showed no effect on the study outcomes but an increase in mortality in babies younger than 32 weeks [64]. Furthermore, studies evaluating safety reported short-term outcomes; thus, long-term follow-up studies should be considered to obtain a better safety evaluation.

## Figures and Tables

**Table 1:** Included studies.

Study	Year	Evaluated INS	Study Characteristics	Main Results
**Animal Studies—Deficiency**				
Hayashi [36]	1974	MI	Rats fed with an MI-deficient diet or the same diet with the addition of 0.5% MI for up to five weeks.	Increased levels of liver TG, CE, and non-esterified fatty acids and concomitant increase in serum non-esterified fatty acids in the MI-deficient group.
Hayashi [37]	1974	MI	Rats fed with an MI-deficient diet or the same diet with the addition of 0.5% MI for one or two weeks.	Liver TG levels were increased in MI-deficient rats, especially in palmitic, palmitoleic, and oleic acids.
Thakur [38]	2011	PI	Zebrafish mutants incapable of PI synthesis.	Mutants exhibited hepatomegaly with microscopic NAFLD features with upregulated endoplasmic reticulum stress markers.
**Animal Studies—Supplementation**				
Katayama [39]	1994	MI	Rats fed with either corn starch or a high-sucrose diet, with or without MI, for 16–17 days.	Reduction in the increase of liver weight, total lipids, TG, and CE by MI in high-sucrose fed rats; reduction in serum TG increase in the same group.
Katayama [40]	1997	MI, sodium phytate	Rats fed with either corn starch or a high-sucrose diet, with or without the addition of MI or sodium phytate for 12–13 days.	MI and sodium phytate reduced liver enlargement and suppressed to normal levels liver TG and total lipids levels; reduced liver G6PD, ME, and FASN.
Geethan [41]	2008	Pinitol	Streptozotocin-induced diabetic rats treated with or without 100 mg/kg Pinitol for 30 days.	Pinitol reduced blood glucose and serum TG, free fatty acids, and CE; decreased TG and CE liver concentration; decreased the concentration of liver phospholipids and free fatty acids; increased HDL and reduced LDL.
Zhou [42]	2008	Pinitol	Rats fed with a high-fat diet for 8 weeks, with or without 0.1%, 1.0%, or 2.0% Pinitol, and induced hepatic injury by a single administration of GalN.	After GalN administration, Pinitol suppressed the increase in ALT and AST; attenuated liver CE increase; reduced TNFα levels; reduced lipid peroxidation; increased glutathione levels; increased liver catalase; Mn–SOD; GR activities.
Choi [43]	2009	Pinitol	Hamsters fed with a high-fat, high-cholesterol diet with or without 0.05% or 0.1% Pinitol for 10 weeks.	Pinitol reduced epididymal and perirenal white adipose tissue; reduced plasma total CE, non-HDL CE, glucose, and total-CE/HDL ratio; reduced liver TG and CE; lowered HMGR and ACAT activities; suppression of liver lipid accumulation and reduction in adipocyte size.
Sivakumar [44]	2010	Pinitol	Streptozotocin-induced diabetic rats treated with Pinitol, gliclazide, or neither for 30 days.	Both Pinitol and gliclazide reversed increase in blood glucose and glycosylated Hgb; reduced blood TNF-α, IL-6, and IL-1β; reduced liver peroxides and hydroperoxides; contrasted the diabetes-induced microscopic liver alterations normalizing the tissue architecture.
Shimada [45]	2019	MI	Rats fed with either a high-glucose or high-fructose diet, with or without MI 0.05% or 0.25% supplementation for 15 days.	MI dose-dependent reduction of liver TG content and expression levels of G6PD, ME1, FASN, ACCα, and S14 in fatty liver high-fructose induced rats; reduction in hepatic ChREBPβ expression; reduction in ChREBP binding to the ChoRE ChREBPβ and FASN genes.
**Human Studies—Supplementation**				
Lee [46]	2019	Pinitol	Double-blind RCT on 90 NAFLD patients taking Pinitol 600 mg, 1000 mg or PBO for 12 weeks	No significant between groups differences in liver fat content at 12 weeks; significant reduction in liver fat content in the 600 mg arm compared to its baseline. Pinitol significantly reduced AST levels at 12 weeks; reduced lipid peroxidation in terms of urinary MDA stability compared to PBO increased GPx. Pinitol reduced blood TG increase after postprandial high-fat formula compared to PBO.

INS = inositol; MI = myoinositol; PI = phosphatidylinositol; TG = triglycerides; GalN = D-galactosamine; ALT = alanine aminotransferase; AST = aspartate aminotransferase; TNFα = tumor necrosis factor alpha; Mn–SOD = MN-superoxide dismutase; GR = glutathione reductase; CE = cholesterol; HMGR = HMG-CoA-Reductase; ACAT = acyl-CoA cholesterol acetyltransferase; Hgb = hemoglobin; G6PD = glucose-6-phosphate-dehydrogenase; ME1 = malic enzyme 1; FASN = fatty acid synthase; ACCα = acetyl-CoA-carboxylase alpha; S14 = modulator of fatty acid synthesis; ChREBP = carbohydrate-responsive element-binding protein; RCT = randomized controlled trial; PBO = placebo; MDA = malondialdehyde; GPx = glutathione peroxidase; non-alcoholic fatty liver disease = NAFLD.

## Supplementary Materials

The following are available online at https://www.mdpi.com/2072-6643/12/11/3379/s1. Suppl.1 Search strategy.
