# Supplementary material for: Inositol and Non-Alcoholic Fatty Liver Disease: A Systematic Review on Deficiencies and Supplementation
Source: Nutrients. 2020 Nov 3;12(11):3379. doi: 10.3390/nu12113379 (PMC7694137; doi:10.3390/nu12113379)
Supplement: Supplementary file 1 [file nutrients-12-03379-s001.pdf]

MEDLINE (Pubmed) (47 up to 09/10/2020)

("Inositol"[MeSH Terms] OR "Inositol"[Title/Abstract] OR "Mesoinositol"[Title/Abstract] OR "Myoinositol"[Title/Abstract] OR "Chiro-inositol"[Title/Abstract] OR "Chiro inositol"[Title/Abstract]) AND ("non alcoholic fatty liver disease"[MeSH Terms] OR "non alcoholic fatty liver disease"[Title/Abstract] OR "NAFLD"[Title/Abstract] OR "Nonalcoholic Fatty Liver Disease"[Title/Abstract] OR "fatty liver nonalcoholic"[Title/Abstract] OR "Nonalcoholic Fatty Liver"[Title/Abstract] OR "Nonalcoholic Fatty Livers"[Title/Abstract] OR "Nonalcoholic Steatohepatitis"[Title/Abstract] OR "steatohepatitis nonalcoholic"[Title/Abstract])

EMBASE (67 up to 09/10/2020)

#1 'nonalcoholic fatty liver'/exp/mj
#2 'non alcoholic fatty liver disease':ti,ab OR nafld:ti,ab OR 'nonalcoholic fatty liver disease':ti,ab OR 'nonalcoholic fatty liver':ti,ab OR 'nonalcoholic fatty livers':ti,ab OR 'nonalcoholic steatohepatiti*':ti,ab
#3 #1 OR #2
#4 'inositol'/exp/mj
#5 'inositol':ti,ab OR mesoinositol:ti,ab OR myoinositol:ti,ab OR 'myo-inositol':ti,ab OR 'chiro inositol':ti,ab
#6 #4 OR #5
#7 #3 AND #6

COCHRANE Library (0 RCTs or Cochrane reviews up to 09/10/2020)

#1 MeSH descriptor: [Non-alcoholic Fatty Liver Disease] explode all trees 930
#2 ("non alcoholic fatty liver disease"):ti,ab,kw OR ("NAFLD"):ti,ab,kw OR ("nonalcoholic fatty liver disease"):ti,ab,kw OR ("nonalcoholic fatty liver*"):ti,ab,kw OR ("Nonalcoholic Steatohepatiti*"):ti,ab,kw (Word variations have been searched) in Cochrane Reviews, Trials 2939
#3 MeSH descriptor: [Inositol] explode all trees 461
#4 ("mesoinositol"):ti,ab,kw OR ("myoinositol"):ti,ab,kw OR ("myo-inositol"):ti,ab,kw OR ("chiroinositol"):ti,ab,kw OR (inositol):ti,ab,kw (Word variations have been searched) in Cochrane Reviews, Trials 791
#5 ("chiro-inositols"):ti,ab,kw OR ("chiro inositol"):ti,ab,kw (Word variations have been searched) in Cochrane Reviews, Trials 78
#6 #1 OR #2 in Cochrane Reviews, Trials (Word variations have been searched) 2939
#7 #3 OR #4 OR #5 in Cochrane Reviews, Trials (Word variations have been searched) 881
#8 #6 AND #7 in Cochrane Reviews, Trials (Word variations have been searched) 0

***Limits applied**: Trials, Cochrane Reviews, All years, All dates, Search word variations*
